# Temporal trends in heart failure mortality in an integrated healthcare delivery system, California, and the US, 2001–2017

**DOI:** 10.1186/s12872-021-02075-6

**Published:** 2021-05-26

**Authors:** Matthew T. Mefford, Zimin Zhuang, Zhi Liang, Wansu Chen, Sandra Y. Koyama, Maria T. Taitano, Heather L. Watson, Ming-Sum Lee, Stephen Sidney, Kristi Reynolds

**Affiliations:** 1 Department of Research and Evaluation, Kaiser Permanente Southern California, 100 S Los Robles Ave, 2nd Floor, Pasadena, CA 91101 USA; 2 Internal Medicine, Kaiser Permanente Southern California, Baldwin Park, CA USA; 3 Harbor City Medical Center, Harbor City, CA USA; 4 Complete Care Support Programs, Kaiser Permanente Southern California, Pasadena, CA USA; 5 Department of Cardiology, Kaiser Permanente Los Angeles Medical Center, Los Angeles, CA USA; 6 Division of Research, Kaiser Permanente Northern California, Oakland, CA USA

**Keywords:** Heart failure, Mortality, Trends, Epidemiology

## Abstract

**Background:**

In recent years, decreases in mortality rates attributable to cardiovascular diseases have slowed but mortality attributable to heart failure (HF) has increased.

**Methods:**

Between 2001–2017, trends in age-adjusted mortality with HF as an underlying cause for Kaiser Permanente Southern California (KPSC) members were derived through linkage with state death files and compared with trends among California residents and the US. Average annual percent change (AAPC) and 95% confidence intervals (CI) were calculated using Joinpoint regression. Analyses were repeated examining HF as a contributing cause of death.

**Results:**

In KPSC, the age-adjusted HF mortality rates were comparable to California but lower than the US, increasing from 23.9 per 100,000 person-years (PY) in 2001 to 44.7 per 100,000 PY in 2017, representing an AAPC of 1.3% (95% CI 0.0%, 2.6%). HF mortality also increased in California from 33.9 to 46.5 per 100,000 PY (AAPC 1.5%, 95% CI 0.3%, 2.7%), while remaining unchanged in the US at 57.9 per 100,000 PY in 2001 and 2017 (AAPC 0.0%, 95% CI − 0.5%, 0.5%). Trends among KPSC members ≥ 65 years old were similar to the overall population, while trends among members 45–64 years old were flat between 2001–2017. Small changes in mortality with HF as a contributing cause were observed in KPSC members between 2001 and 2017, which differed from California and the US.

**Conclusion:**

Lower rates of HF mortality were observed in KPSC compared to the US. Given the aging of the US population and increasing prevalence of HF, it will be important to examine individual and care-related factors driving susceptibility to HF mortality.

**Supplementary Information:**

The online version contains supplementary material available at 10.1186/s12872-021-02075-6.

Heart failure (HF) is a major public health problem that is associated with high hospital readmission rates, mortality, and health care expenditures [1]. Despite treatment advances, mortality remains high with approximately 50% of HF patients dying within 5 years of their diagnosis [1]. Overall, 1 in 8 deaths list HF as a contributing cause [2]. While all-cause mortality and the mortality attributable to cardiovascular disease (CVD) have declined over the past decades, declines in rates of CVD mortality began slowing in 2011 [3–9]. Mortality attributable to HF, in contrast, began increasing around this time [4, 10]. Explanations for the increase in HF mortality may include the aging population, the increasing prevalence of comorbidities, and the growing prevalence of HF with preserved ejection fraction, which lacks specific evidence-based treatments [11–14]. Examining temporal trends in HF mortality is important as this may reflect changes in individual, or institutional factors, and can inform healthcare policy and practice [15]. Integrated healthcare delivery systems are associated with better adherence to evidence-based care guidelines, survival rates, and reduced racial disparities [16–18]. Kaiser Permanente Southern California (KPSC) serves approximately 4.6 million enrollees and largely reflects the diverse population of southern California, making it ideal to study mortality trends [19]. Therefore the purpose of the current study was to examine secular trends in HF mortality among adults ≥ 45 years old in KPSC, California, and the US, from 2001 to 2017.

## Methods

### Study population

KPSC is an integrated healthcare delivery system that serves approximately 20% of the Southern California population. At enrollment into the health plan, date of birth and sex were collected administratively. Race/ethnicity was based on a combination of administrative and patient self-report as previously described [20]. Health plan enrollees 45–110 years old with enrollment between 2001 and 2017 were included in the current analysis. For each member, the total number of days enrolled was considered the at-risk time for each year. At-risk time for each year began on the date of KPSC enrollment or January 1, whichever occurred later, and ended at disenrollment from the KPSC health plan, date of death, or December 31, whichever occurred first. Gaps in enrollment ≤ 45 days, including those that spanned 2 consecutive years, were bridged (i.e., continuous enrollment was assumed). Members with missing information on date of birth or sex labeled as “other” or “unknown” were excluded. Enrollees with race/ethnicity other than non-Hispanic white, non-Hispanic black, Hispanic, and Asian/Pacific Islander, or who had missing information on race were included in analyses not specific to race/ethnicity. The study protocol was approved by the KPSC institutional review board.

### Mortality

HF as an underlying and, separately, contributing cause of death was identified among eligible adults. The underlying cause of death is defined as the disease or injury that initiates the chain of morbid events leading directly to death [21]. Contributing causes of death are defined as all significant diseases, conditions, or injuries that contributed to death but which did not result in the underlying cause of death. KPSC death records were derived by identifying deaths that occurred at KPSC-owned facilities (i.e., patient electronic health records), outside facilities that submitted claims to KPSC, or deaths reported to the health plan. Additionally, death records were supplemented by linking members with decedents in the California Death Statistical Master Files, the California Comprehensive Death File, the State Multiple Cause of Death File, and the Social Security Administration (SSA) Death Master Files. Linkage of the death files to members in KPSC has been described previously [15]. Causes of death were determined by International Classification of Diseases, Clinical Modification, 10th Revision (ICD-10-CM) codes in the California State death files. US and California mortality rates between 2001 and 2017 for adults ≥ 45 years old were derived from the Center for Disease Control’s (CDC) Wide-Ranging Online Data for Epidemiologic Research CDC WONDER dataset, which includes underlying and contributing causes of death from death certificates filed in the 50 states and the District of Columbia [2, 22]. Underlying cause of death from HF was identified with ICD-10 code I50.x. HF as a contributing cause of death was defined as mortality with an underlying cardiovascular cause (ICD-10 codes I00-I78) with HF listed as a contributing cause [23].

### Statistical analysis

Age, sex, and race/ethnicity were described overall and for each year from 2001 to 2017. Overall, sex-stratified, and race-stratified age-adjusted mortality rates per 100,000 person-years (PY) were calculated using the direct method with the 2000 US Census population as the standard population. US Census counts are reported in 5-year age intervals [24].

Average annual percentage changes (AAPC) in mortality rates and 95% confidence intervals (CI) were estimated using the Joinpoint Regression Program, Version 4.5.0.1 (Statistical Research and Applications Branch, National Cancer Institute) [25]. To compare AAPCs in KPSC with California and the US, we calculated a z-statistic by dividing the difference in the two AAPCs (KPSC vs California; or KPSC vs US) and the standard error (SE) of the difference (i.e., square root [SE1^2^ + SE2^2^], where SE1 and SE2 are the standard errors of the two individual estimates). The analysis was performed on a log scale, and conducted overall and by age and sex groups, separately. We additionally calculated HF mortality rates within race/ethnicity groups (white, black, Hispanic, Asian/Pacific Islander [API]) over the same study period.

Given evidence of increasing rates of HF mortality since 2011 [10], we also examined trends from 2001–2011 and 2011–2017, separately. Using best-fit Joinpoint models for the entire study period (2001–2017) period-specific AAPCs for 2001–2011 and 2011–2017 were calculated [25]. Best-fit models were selected by permutation testing that identified the optimal number of knots [26]. We repeated the above analyses to examine mortality with HF listed as a contributing cause of death overall and by sex. All tests were two-sided and *p* < 0.05 was considered statistically significant.

## Results

Within KPSC, members ≥ 45 years old increased from 0.95 million in 2001 to approximately 1.78 million in 2017. (Table 1) From 2001 to 2017, the proportion of adults 45–64 years old decreased (69.0% to 63.8%) while the proportion of adults ≥ 65 years old increased (31.0% to 36.2%). The proportions of white and black adults decreased (55.3% to 43.4% and 12.1% to 9.7%, respectively) while the proportions of Hispanic and API adults increased (23.8% to 34.8% and 8.9% to 12.2%, respectively).

**Table 1:** Demographic characteristics of Kaiser Permanente Southern California adult enrollees ≥ 45 years of age, 2001–2017

	2001	2002	2003	2004	2005	2006	2007	2008	2009	2010	2011	2012	2013	2014	2015	2016	2017
Enrollees, n	945,676	996,689	1,030,657	1,059,319	1,104,743	1,157,917	1,201,172	1,235,241	1,276,507	1,330,215	1,389,925	1,448,424	1,493,217	1,569,391	1,666,634	1,726,153	1,783,391
Deaths, %	1.3	1.3	1.3	1.3	1.3	1.2	1.2	1.1	1.1	1.1	1.11	1.1	1.2	1.1	1.2	1.2	1.2
Age, years, %																	
45–54	41.0	40.6	40.0	39.5	39.3	39.4	39.4	39.1	38.2	37.2	36.4	35.3	34.3	33.8	33.7	33.2	32.6
55–64	28.0	28.7	29.2	29.6	29.9	30.4	30.8	31.1	31.5	31.9	32.0	31.5	31.3	31.2	31.3	31.2	31.2
65–74	18.7	18.4	18.4	18.4	18.1	17.7	17.4	17.4	17.7	18.1	18.7	19.9	20.9	21.3	21.4	21.9	22.2
75–84	9.8	9.8	9.8	9.8	9.8	9.6	9.5	9.3	9.4	9.5	9.5	9.7	10.0	10.0	9.9	10.0	10.2
85 +	2.5	2.6	2.7	2.7	2.8	2.9	3.0	3.1	3.2	3.3	3.4	3.5	3.6	3.6	3.7	3.8	3.8
Male, %	46.5	46.4	46.4	46.4	46.5	46.5	46.6	46.6	46.6	46.6	46.6	46.7	46.7	46.7	46.8	46.8	46.7
Race/Ethnicity*, %																	
API	8.9	9.1	9.3	9.5	9.7	9.9	10.1	10.4	10.6	10.8	11.0	11.1	11.2	11.4	11.6	11.8	12.2
Black	12.1	11.8	11.7	11.7	11.5	11.3	11.2	11.1	11.0	10.8	10.7	10.5	10.4	10.2	10.0	9.8	9.7
Hispanic	23.8	24.7	25.3	26.0	27.0	28.1	28.9	29.6	29.9	30.0	30.3	30.8	31.4	32.5	33.4	34.2	34.8
NH White	55.3	54.3	53.7	52.9	51.8	50.8	49.8	49.0	48.6	48.4	48.1	47.7	47.0	45.9	45.0	44.1	43.4

*API* Asian/Pacific Islander; *NH White* Non-Hispanic White

^*^Among members with known race/ethnicity

### HF as an underlying cause of death

**Fig. 1:**
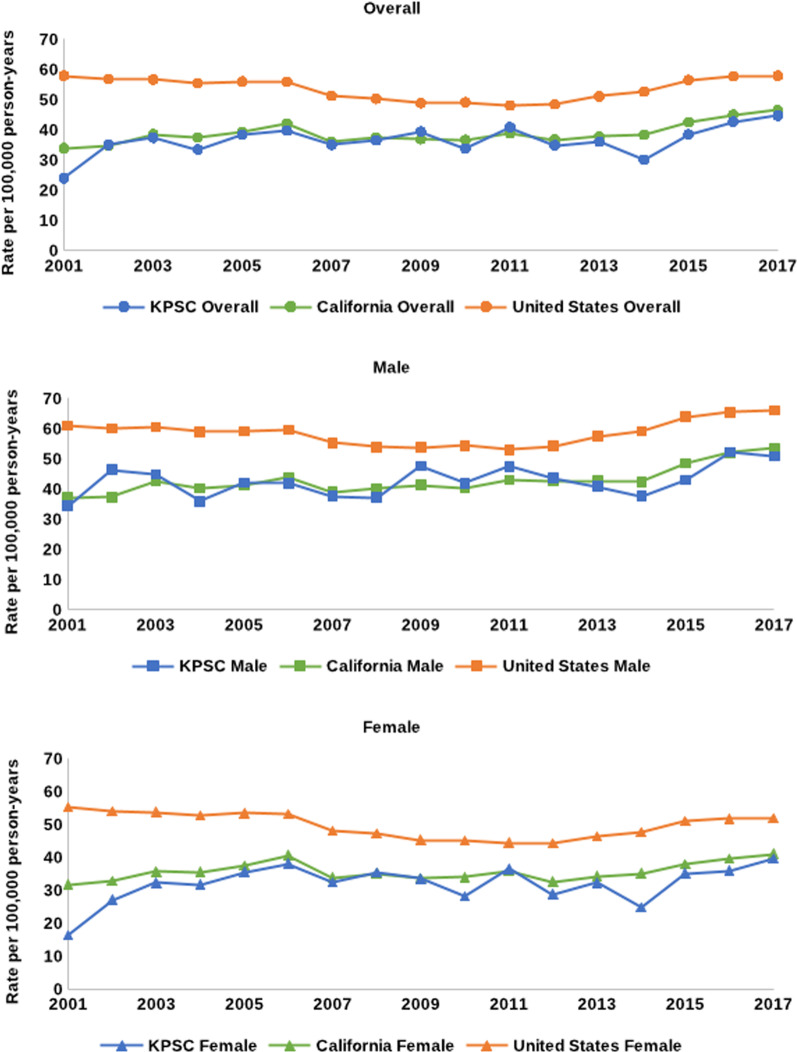
Mortality with heart failure as an underlying cause in KPSC, California, and the US, 2001–2017

Between 2001 and 2017, KPSC rates of mortality with HF as an underlying cause were similar to California but lower compared to the US. In KPSC, the age-adjusted mortality rate with HF as the underlying cause increased from 23.9 per 100,000 PY in 2001 to 44.7 per 100,000 PY in 2017, representing an AAPC of 1.3% (95% CI 0.0%, 2.6%). (Fig. 1 and Table 2) During the same time period, HF mortality rates also increased in California from 33.9 to 46.5 per 100,000 PY (AAPC 1.5%, 95% CI 0.3%, 2.7%), while remaining unchanged in the US at 57.9 per 100,000 PY in 2001 and 2017 (AAPC 0.0%, 95% CI − 0.5%, 0.5%). From 2001–2017, HF mortality trends were not different comparing KPSC to California and the US (all *p*-values > 0.05 [data not shown]). In KPSC, the AAPCs for women and men were 4.1% (95% CI − 3.7%, 12.6%) and 1.3% (95% CI 0.1%, 2.5%), respectively, between 2001 and 2017. Sex-specific trends from 2001 to 2017 were not statistically different from those in California and the US. In KPSC, HF mortality increased at the same rate from 2001 to 2011 and 2011 to 2017. In contrast, HF mortality rates remained flat in California and in the US from 2001 to 2011 while significantly increasing from 2011 to 2017.

**Table 2:** Mortality with HF as the underlying cause in KPSC, California, and the United States, 2001–2017

Year	KPSC			California			United States		
	Male	Female	Overall	Male	Female	Overall	Male	Female	Overall
2001	34.4	16.4	23.9	37.2	31.7	33.9	60.9	55.3	57.9
2002	46.4	27.1	35.0	37.3	32.8	34.7	60.1	54.0	56.8
2003	44.9	32.3	37.3	42.5	35.7	38.5	60.5	53.7	56.6
2004	35.9	31.8	33.5	40.2	35.5	37.5	59.0	52.8	55.5
2005	42.1	35.5	38.3	41.2	37.5	39.2	59.1	53.5	56.0
2006	41.9	38.1	39.7	43.7	40.7	42.1	59.5	53.1	55.9
2007	37.6	32.5	35.0	39.0	33.9	36.0	55.2	48.2	51.2
2008	37.2	35.5	36.5	40.3	35.1	37.4	53.9	47.3	50.3
2009	47.7	33.5	39.4	41.4	33.7	36.9	53.7	45.2	48.8
2010	41.9	28.2	33.8	40.3	34.0	36.6	54.4	45.2	49.1
2011	47.4	36.6	40.8	43.0	35.8	38.8	53.0	44.4	48.1
2012	43.6	28.8	34.8	42.7	32.5	36.7	54.2	44.2	48.5
2013	40.9	32.3	36.0	42.7	34.2	37.7	57.4	46.4	51.1
2014	37.6	24.9	30.1	42.4	35.1	38.3	59.2	47.8	52.7
2015	43.0	35.1	38.5	48.5	38.0	42.5	63.9	51.0	56.5
2016	52.2	36.0	42.7	52.0	39.6	45.0	65.4	51.8	57.7
2017	50.8	39.6	44.7	53.5	41.0	46.5	66.0	51.9	57.9
AAPC (95% CI)									
Overall	1.3* (0.1, 2.5)	4.1 (−3.7, 12.6)	1.3* (0.0, 2.6)	2.2* (1.2, 3.1)	1.6 (−0.1, 3.4)	1.5* (0.3, 2.7)	0.5* (0.1, 1.0)	−0.2 (−1.0, 0.6)	0.0 (−0.5, 0.5)
2001–2011	1.3* (0.1, 2.5)	6.5 (−5.9, 20.5)	1.3* (0.0, 2.6)	0.8* (0.2, 1.5)	0.6 (−1.7, 2.9)	0.2 (−0.8, 1.2)	−1.6 * (−2.1, 1.0)	−2.3 * (−3.4, − 1.2)	−2.1* (−2.7, − 1.5)
2011–2017	1.3* (0.1, 2.5)	0.3 (−1.6, 2.2)	1.3* (0.0, 2.6)	4.4* (2.2, 6.7)	3.5* (1.1, 5.9)	3.7* (1.0, 6.5)	4.1* (3.1, 5.2)	3.4* (2.2, 4.6)	3.6* (2.4, 4.8)

*AAPC* average annual percent change; *CI* confidence interval; *HF* heart failure; *KPSC* Kaiser Permanente Southern California

Rates of mortality presented as per 100,000 person-years and age-adjusted

^*^denotes *p* < 0.05

Among members 45–64 years old, between 2001 and 2017 the age-adjusted mortality rate with HF as the underlying cause increased from 1.1 to 2.2 per 100,000 PY in KPSC, representing an AAPC of 1.6% (95% CI − 1.6%, 4.9%). (Additional file 1: Table 1) During this same period, HF mortality rates also increased in California from 2.6 to 4.7 per 100,000 PY (AAPC 3.1%, 95% CI 0.7%, 5.6%) and in the US from 5.2 to 6.4 per 100,000 PY (AAPC 1.6%, 95% CI 1.0%, 2.2%). In KPSC, the AAPCs for women and men 45–64 years old were 2.3% (95% CI − 3.3%, 8.3%) and 1.0% (95% CI − 1.3% to 3.3%), respectively, between 2001 and 2017. Overall and sex-specific HF mortality trends from 2001 to 2017 were not different comparing KPSC to California and the US (all *p*-values > 0.05 [data not shown]). In KPSC, age-adjusted HF mortality increased at the same rate from 2001 to 2011 and 2011 to 2017. In contrast, HF mortality rates remained flat in CA and in the US from 2001 to 2011 while significantly increasing from 2011 to 2017.

Among members ≥ 65 years old in KPSC, between 2001 and 2017 the age-adjusted mortality rate with HF as the underlying cause of death increased from 64.0 per 100,000 PY in 2001 to 119.4 per 100,000 PY in 2017, representing an AAPC of 1.3% (95% CI 0.0%, 2.5%). (Additional file 1: Table 2) During this same period, HF mortality rates also increased in California from 89.0 to 119.8 per 100,000 PY (AAPC 1.5%, 95% CI 0.2%, 2.8%) but were higher and decreased slightly in the US from 150.6 to 148.4 per 100,000 PY (AAPC − 0.1%, 95% CI − 0.6%, 0.4%). HF mortality trends from 2001 to 2017 were not different comparing KPSC to California; however, mortality trends comparing KPSC to the US were statistically different (*p* = 0.05 [data not shown]). In KPSC, the AAPCs for women and men ≥ 65 years old were 5.3% (95% CI − 0.3%, 11.3%) and 1.3% (95% CI 0.1%, 2.5%), respectively, between 2001 and 2017. The AAPC among women in KPSC was statistically different from that of the US (5.3% vs − 0.1%, *p* = 0.05 [data not shown]). No differences were detected comparing KPSC men to California or the US. In KPSC members, age-adjusted HF mortality increased at the same rate from 2001 to 2011 and 2011 to 2017. In contrast, HF mortality rates remained flat in CA and decreased in the US from 2001 to 2011 while significantly increasing in both populations from 2011 to 2017.

Among KPSC enrollees in 2017, API members had the lowest age-adjusted HF mortality (24.8 per 100,000 PY) followed by Hispanic (31.4 per 100,000 PY), white (52.0 per 100,000 PY) and black (53.2 per 100,000 PY) members. (Additional file 1: Table 3) Rates of HF mortality were similar among API but lower among white, Hispanic, and black individuals in KPSC compared to the US. Race/ethnicity-specific variations in HF mortality rates between KPSC, California, and the US were also observed among adults 45–64 years old (Additional file 1: Table 4) and adults ≥ 65 years old (Additional file 1: Table 5).

**Table 3:** Mortality with HF as a contributing cause in KPSC, California, and the United States, 2001–2017

Year	KPSC			California			United States		
	Male	Female	Overall	Male	Female	Overall	Male	Female	Overall
2001	188.0	110.7	143.3	232.7	178.7	201.2	232.9	187.2	206.2
2002	190.4	122.6	150.7	233.2	174.9	199.2	228.4	181.2	200.5
2003	183.0	135.1	155.9	231.5	176.0	199.2	223.8	178.3	197.0
2004	192.1	121.7	151.6	220.2	166.9	189.1	215.6	170.5	189.0
2005	198.5	136.7	163.0	220.2	166.2	188.8	214.8	171.3	189.1
2006	188.9	131.6	156.0	211.1	158.7	180.6	203.1	159.5	177.5
2007	179.7	127.8	150.0	193.6	146.6	166.4	192.9	150.6	168.1
2008	175.2	120.0	143.3	192.3	143.4	163.9	188.5	146.6	164.0
2009	181.7	115.0	143.0	184.7	133.7	155.0	182.1	138.1	156.3
2010	175.5	112.1	138.8	181.1	130.9	151.7	181.7	136.3	155.0
2011	172.9	119.6	142.4	178.1	129.8	150.1	176.7	133.3	151.3
2012	164.0	110.6	133.2	173.4	121.4	143.0	176.5	131.0	149.9
2013	171.2	113.5	137.4	176.3	121.2	144.2	181.3	132.9	153.1
2014	162.5	102.7	127.8	167.5	117.2	138.6	183.1	133.5	154.4
2015	163.7	113.6	135.1	177.8	123.8	146.8	190.1	140.2	161.4
2016	166.7	112.0	135.3	182.0	126.2	150.1	191.7	138.3	160.9
2017	170.3	117.0	139.9	183.5	126.6	151.2	194.2	139.3	162.7
AAPC (95% CI)									
Overall	−1.0* (−1.4, − 0.7)	0.3 (−1.8, 2.5)	−0.3 (−1.2, 0.6)	−1.7* (−2.3, − 1.2)	−2.1* (−3.1, − 1.1)	−1.7* (−2.7, − 0.8)	−1.2* (−1.5, − 0.9)	−1.9* (−2.3, − 1.5)	−1.5* (−1.9, − 1.2)
2001–2011	− 1.0* (− 1.4, − 0.7)	0.3 (− 2.7, 3.4)	− 0.5 (− 1.6, 0.5)	− 3.1* (− 3.6, − 2.6)	− 3.3* (− 4.5, − 2.1)	− 3.0* (− 4.2, − 1.9)	− 3.0* (− 3.3, − 2.6)	− 3.7* (− 4.1, − 3.2)	− 3.3* (− 3.7, − 3.0)
2011–2017	− 1.0* (− 1.4, − 0.7)	0.4 (− 2.2, 3.0)	0.1 (− 1.3, 1.6)	0.6 (− 0.6, 1.8)	0.1 (− 1.4, 1.6)	0.5 (− 1.0, 1.9)	1.9* (1.2, 2.6)	1.1* (0.1, 2.0)	1.5* (0.8, 2.3)

*AAPC* average annual percent change; *CI* confidence interval; *HF* heart failure; *KPSC* Kaiser Permanente Southern California

Rates of mortality presented as per 100,000 person-years and age-adjusted

^*^denotes *p* < 0.05

### HF as a contributing cause of death

**Fig. 2:**
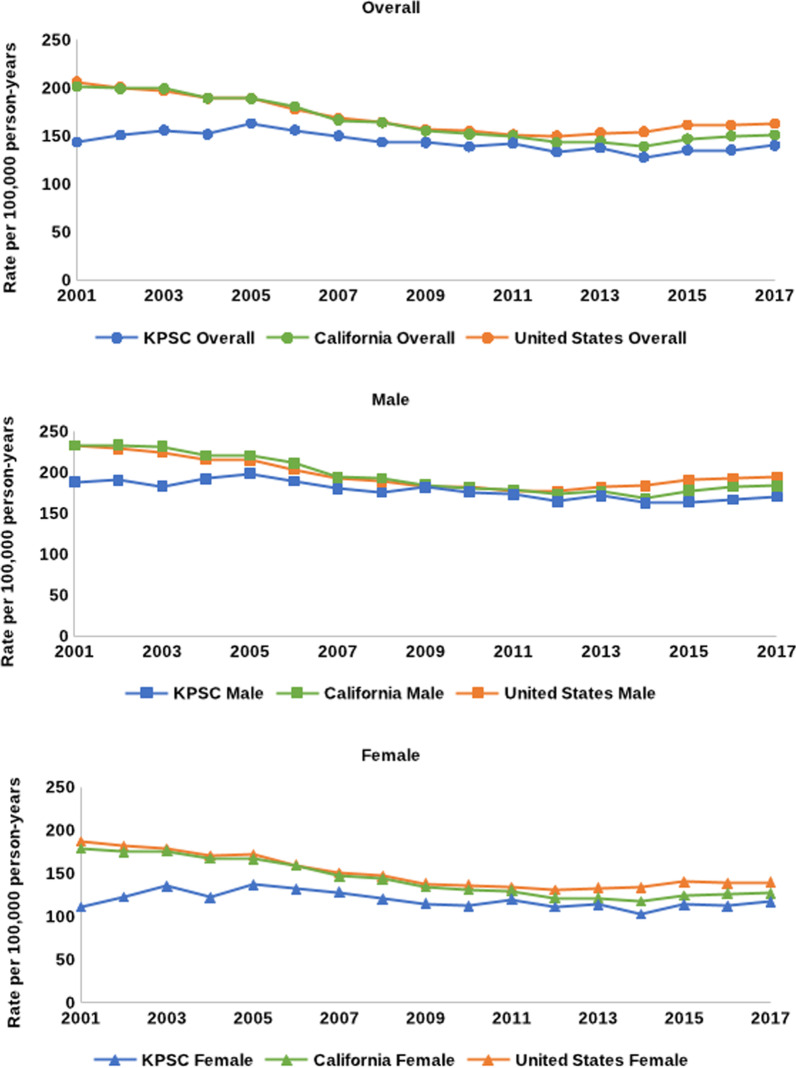
Mortality with heart failure as a contributing cause in KPSC, California, and the US, 2001–2017

Between 2001 and 2017, age-adjusted mortality with HF as a contributing cause followed a similar pattern to mortality with HF as an underlying cause. (Fig. 2) In KPSC, rates changed from 143.3 per 100,000 PY in 2001 to 139.9 per 100,000 PY in 2017, representing an AAPC of − 0.3% (95% CI − 1.2%, 0.6%). (Table 3) During the same time period, rates were higher but also changed in California from 201.2 to 151.2 per 100,000 PY (AAPC − 1.7%, 95% CI − 2.7%, − 0.8%) and in the US from 206.2 to 162.7 per 100,000 PY (AAPC − 1.5%, 95% CI − 1.9%, − 1.2%). Overall trends were different comparing KPSC to California and the US (*p*-value 0.04 and 0.02, respectively, [data not shown]). In KPSC, from 2001 to 2017 the AAPCs for women and men were 0.3% (95% CI − 1.8%, 2.5%) and − 1.0% (95% CI − 1.4%, − 0.7%), respectively. Trends in KPSC compared to both California and the US were different among women (both *p* = 0.04) but not for men (*p* = 0.06 and *p* = 0.43, respectively, [data not shown]). Between the time periods 2001–2011 and 2011–2017, mortality rates with HF as a contributing cause remained flat in both KPSC and California; in the US, rates decreased from 2001 to 2011 and subsequently increased from 2011 to 2017.

## Discussion

In the current study, HF mortality rates in KPSC were similar to California but lower than the US, with rates increasing from 2001 to 2017. This was observed in KPSC men but not among women. Trends among KPSC members ≥ 65 years old increased as in the overall population, while no change in HF mortality rates were observed in KPSC members 45–64 years old. Mortality with HF as a contributing cause decreased significantly among KPSC men, but not women. For HF mortality as both an underlying and contributing cause, smaller changes in KPSC compared to California and the US were observed between older (2001–2011) and contemporary (2011–2017) time periods.

HF mortality rates were lower in KPSC compared to the US, and the magnitude of differences in mortality rates between KPSC and the US decreased between 2001 and 2017. Overall, US rates in the current study fluctuated between 48 and 58 per 100,000 PY for all adults ≥ 45 years old, consistent with previous studies. An analysis of data from the National Center for Health Statistics (NCHS) reported 2015 age-adjusted mortality rates with HF as the underlying cause of death ranging from 1.7 per 100,000 PY among US adults 40–49 years old to 441.1 per 100,000 PY among those ≥ 80 years old [27]. While the age compositions of populations in each study may account for the variability of rates, the current study results are similar in magnitude to observed US rates. Reasons for lower HF mortality rates in KPSC, particularly in 2001, may require additional investigation. However, this may be partially attributable to better treatment and management of other preventable causes of death; a previous KPSC study noted that HD mortality decreased between 2001 and 2016 [15]. Lower rates of HF mortality compared to the US may also be attributable to the KPSC integrated healthcare delivery model. KPSC has previously noted improvements in HF risk factors, including blood pressure and lipids [28, 29]. Additionally, while KPSC is representative of the Southern California population, some differences in mortality rates may be explained by the sociodemographic differences between KPSC, California, and the US.

HD mortality, and more specifically, mortality attributable to HF began slowing around 2011, and even increasing more recently [4, 6, 10]. Sidney and colleagues examined US mortality trends attributable to all HD from 2000 to 2015, noting decreasing rates of HF mortality between 2000 and 2011 but annual increases between 2011 and 2015 by 3.73% (95% CI 3.21%, 4.26%) [10] An additional study by Sidney reported this trend continued through 2017, and represented a 38% increase in deaths with HF listed as an underlying cause between 2011 and 2017 [14]. Similar trends were reported using NCHS data, where age-adjusted HF mortality decreased between 2000 and 2012 but increased between 2012 and 2014 [4]. In KPSC, we noted that mortality with HF as an underlying cause increased consistently between 2001 and 2017, which contrasts with the larger AAPCs in California and the US between 2011 and 2017. We did not examine factors contributing to these increases. However, increased awareness of HF as a diagnosis may contribute to the increased designation of HF as the underlying cause of death.

Sex-based differences in HF mortality were noted among adults ≥ 65 years old in KPSC, where women compared with men had a larger AAPC. This is consistent with data from California but differs from that of the US. One explanation could be the higher rates of HF mortality in the US compared to KPSC and California at the beginning of the study period; although it is important to note that an increasing AAPC was noted for HF mortality among older adults from 2011–2017. We also note some differences between KPSC, California, and the US with respect to race/ethnicity. However, we interpret these results cautiously due to small sample sizes within subgroups. Similar to this study, Sidney and colleagues noted differences in annual declines of HF mortality in 2000–2011 and 2011–2015 among sex and race/ethnicity groups [10]. In contrast, an analysis of the National Inpatient Sample between 1991 and 2015 reported HF mortality rates varied by race/ethnicity but not sex [27].

Previous studies using national data have reported declines in mortality with HF as a contributing cause. Glynn and colleagues reported a decline through 2011–2012, but an increase through 2012–2017, while a separate study reported a decline through 2014 [4, 23]. Reasons why declines in HF mortality are observed through 2011 may include the emphasis of blood pressure and cholesterol management, and increased smoking cessation. Sustained increases in comorbidities such as obesity and diabetes, reduced mortality from other forms of CVD, and the growth of the population ≥ 65 years old may explain more recent increases in HF mortality [10, 14, 15, 30, 31]. Emphasis on treating and managing HF and accompanying risk factors may help reduce these increasing rates of mortality.

We acknowledge some limitations. HF is considered a mediator between various disease states and mortality, and HF listed as an underlying cause of death may be attributable to other conditions [1]. Thus, ICD-10 coding guidelines suggest listing other plausible heart conditions as the underlying cause of death and listing HF when the etiology cannot be determined. Regardless, we observed similar patterns in mortality with HF listed as a contributing cause of death. Additionally, deaths among KPSC enrollees occurring outside of California may not be completely captured. After 2011, a law was established that prevented the SSA from disclosing state death records received through its contracts. However, it was assumed that most deaths outside of the state of California were reported to KPSC by family, employers, caregivers, doctors outside of the state, and law enforcement. Additionally, cause of death was not available on all enrollees, which may underestimate HF-specific mortality. In the current study, we noted small numbers of HF mortality among stratified subgroups by younger age (45–64 years) and race/ethnicity, which limits the interpretability of our findings. Rates of mortality in the KPSC population may not be generalizable to uninsured populations. Additionally, race/ethnicity was not available for all members. Finally, factors associated with HF mortality were not examined, and this warrants further investigation. Prior work has shown that patient self-care (e.g., weight monitoring, medication adherence, healthy lifestyle behaviors, and comorbidity management) and other care-related factors (e.g., patient education and connection with care) are important in the prognosis of HF patients. [32–34]

In conclusion, lower rates of HF mortality were observed in KPSC compared to the US. Given the aging of the US population and increasing prevalence of HF, it will be important to examine individual and care-related factors driving susceptibility to HF mortality.

## Supplementary Information

**Additional file 1.** **Supplemental Table 1.** Age-adjusted mortality rates (per 100,000 person-years) with heart failure as the underlying cause of death by sex among adults 45–64 years of age in Kaiser Permanente Southern California, California, and the United States, 2001–2017. **Supplemental Table 2.** Age-adjusted mortality rates (per 100,000 person-years) with heart failure as the underlying cause of death by sex among adults ≥65 years of age in Kaiser Permanente Southern California, California, and the United States, 2001–2017. **Supplemental Table 3.** Age-adjusted mortality rates (per 100,000 person-years) with heart failure as the underlying cause of death by sex and race/ethnicity among ≥ 45 years of age in Kaiser Permanente Southern California members, California, and the United States, 2001–2017. **Supplemental Table 4.** Age-adjusted mortality rates (per 100,000 person-years) with heart failure as the underlying cause of death among adults 45-64 years of age by sex and race/ethnicity among Kaiser Permanente Southern California members, California, and the United States, 2001–2017. **Supplemental Table 5.** Age-adjusted mortality rates (per 100,000 person-years) with heart failure as the underlying cause of death among adults ≥ 65 years of age by sex and race/ethnicity among Kaiser Permanente Southern California members, California, and the United States, 2001–2017.

## Data Availability

Anonymized data that support the findings of this study may be made available from the investigative team in the following conditions: (1) agreement to collaborate with the study team on all publications, (2) provision of external funding for administrative and investigator time necessary for this collaboration, (3) demonstration that the external investigative team is qualified and has documented evidence of training for human subjects protections, and (4) agreement to abide by the terms outlined in data use agreements between institutions.
