# Supplementary material for: Temporal trends in heart failure mortality in an integrated healthcare delivery system, California, and the US, 2001–2017
Source: BMC Cardiovasc Disord. 2021 May 26;21:261. doi: 10.1186/s12872-021-02075-6 (PMC8157708; doi:10.1186/s12872-021-02075-6)
Supplement: Supplementary file 1 — Additional file 1. Supplemental Table 1. Age-adjusted mortality rates (per 100,000 person-years) with heart failure as the underlying cause of death by sex among adults 45–64 years of age in Kaiser Permanente Southern California, California, and the United States, 2001–2017. Supplemental Table 2. Age-adjusted mortality rates (per 100,000 person-years) with heart failure as the underlying cause of death by sex among adults ≥65 years of age in Kaiser Permanente Southern California, California, and the United States, 2001–2017. Supplemental Table 3. Age-adjusted mortality rates (per 100,000 person-years) with heart failure as the underlying cause of death by sex and race/ethnicity among ≥ 45 years of age in Kaiser Permanente Southern California members, California, and the United States, 2001–2017. Supplemental Table 4. Age-adjusted mortality rates (per 100,000 person-years) with heart failure as the underlying cause of death among adults 45-64 years of age by sex and race/ethnicity among Kaiser Permanente Southern California members, California, and the United States, 2001–2017. Supplemental Table 5. Age-adjusted mortality rates (per 100,000 person-years) with heart failure as the underlying cause of death among adults ≥ 65 years of age by sex and race/ethnicity among Kaiser Permanente Southern California members, California, and the United States, 2001–2017. [file 12872_2021_2075_MOESM1_ESM.docx]

Online Data Supplement

Trends in Heart Failure Mortality in an Integrated Healthcare Delivery System, California, and the US, 2001-2017

Matthew T. Mefford^a^, PhD, Zimin Zhuang^a^, MS, Zhi Liang^a^, PhD, Wansu Chen^a^, PhD, Sandra Y. Koyama^b^, MD, Maria T. Taitano^c^, MD, Heather L. Watson^d^, MBA/HCM, Ming-Sum Lee^e^, MD, PhD, Stephen Sidney^f^, MD, MPH, Kristi Reynolds^a^, PhD

^a^Department of Research & Evaluation, Kaiser Permanente Southern California, Pasadena, CA

^b^Internal Medicine, Kaiser Permanente Southern California, Baldwin Park, CA

^c^Harbor City Medical Center, Harbor City, CA

^d^Complete Care Support Programs, Kaiser Permanente Southern California, Pasadena, CA

^e^Department of Cardiology, Kaiser Permanente Los Angeles Medical Center, Los Angeles, CA

^f^Division of Research, Kaiser Permanente Northern California, Oakland, CA

Address correspondence and reprint requests to: Matthew Mefford, PhD, Department of Research and Evaluation, Kaiser Permanente Southern California, 100 S Los Robles Ave, 2^nd^ Floor, Pasadena, CA 91101; Email: [matthew.t.mefford@kp.org](mailto:matthew.t.mefford@kp.org); Phone: 626-564-5950

Supplemental Table 1. Age-adjusted mortality rates (per 100,000 person-years) with heart failure as the underlying cause of death by sex among adults 45-64 years of age in Kaiser Permanente Southern California, California, and the United States, 2001-2017

| Year | KPSC | | | California | | | United States | | |
| --- | --- | --- | --- | --- | --- | --- | --- | --- | --- |
|  | Male | Female | Overall | Male | Female | Overall | Male | Female | Overall |
| 2001 | 1.7 | 0.6 | 1.1 | 3.2 | 2.1 | 2.6 | 6.2 | 4.2 | 5.2 |
| 2002 | 3.5 | 1.7 | 2.5 | 3.5 | 2.2 | 2.8 | 6.0 | 3.9 | 5.0 |
| 2003 | 2.7 | 0.3 | 1.4 | 3.9 | 2.3 | 3.1 | 6.0 | 3.9 | 4.9 |
| 2004 | 2.6 | 1.6 | 2.0 | 3.6 | 2.4 | 3.0 | 5.8 | 3.9 | 4.8 |
| 2005 | 2.2 | 1.2 | 1.7 | 3.4 | 2.0 | 2.7 | 5.9 | 3.9 | 4.9 |
| 2006 | 3.2 | 2.4 | 2.8 | 4.9 | 3.1 | 3.9 | 6.5 | 4.0 | 5.2 |
| 2007 | 2.3 | 0.2 | 1.2 | 3.4 | 2.1 | 2.7 | 5.9 | 3.7 | 4.7 |
| 2008 | 3.0 | 2.2 | 2.6 | 3.7 | 2.2 | 3.0 | 6.1 | 3.5 | 4.8 |
| 2009 | 1.9 | 0.4 | 1.1 | 3.8 | 1.9 | 2.8 | 6.1 | 3.5 | 4.8 |
| 2010 | 2.2 | 1.4 | 1.8 | 3.4 | 2.0 | 2.7 | 5.9 | 3.5 | 4.7 |
| 2011 | 3.2 | 1.3 | 2.2 | 3.6 | 2.0 | 2.8 | 5.6 | 3.5 | 4.5 |
| 2012 | 2.3 | 0.8 | 1.5 | 3.8 | 2.1 | 2.9 | 6.0 | 3.5 | 4.7 |
| 2013 | 3.4 | 1.9 | 2.6 | 4.5 | 2.2 | 3.4 | 6.7 | 3.8 | 5.3 |
| 2014 | 1.7 | 0.7 | 1.2 | 4.8 | 2.8 | 3.8 | 7.0 | 4.2 | 5.6 |
| 2015 | 2.8 | 1.8 | 2.3 | 6.0 | 3.0 | 4.5 | 7.7 | 4.3 | 5.9 |
| 2016 | 3.0 | 2.8 | 2.9 | 6.2 | 3.1 | 4.6 | 8.0 | 4.7 | 6.3 |
| 2017 | 3.4 | 1.0 | 2.2 | 6.7 | 2.9 | 4.7 | 8.4 | 4.6 | 6.4 |
| AAPC (95% CI) | | | | | | | | | |
| Overall | 1.0 (-1.3, 3.3) | 2.3 (-3.3, 8.3) | 1.6 (-1.6, 4.9) | 3.9* (1.6, 6.2) | 1.8 (-1.2, 4.9) | 3.1* (0.7, 5.6) | 2.0* (1.4, 2.7) | 0.9* (0.2, 1.6) | 1.6*  (1.0, 2.2) |
| 2001-2011 | 1.0 (-1.3, 3.3) | 2.3 (-3.3, 8.3) | 1.6 (-1.6, 4.9) | -0.1 (-2.9, 2.8) | -1.4 (-4.9, 2.2) | -0.6 (-3.5, 2.4) | -0.5 (-1.3, 0.3) | -1.9* (-2.7, -1.1) | -1.0*  (-1.7, -0.4) |
| 2011-2017 | 1.0 (-1.3, 3.3) | 2.3 (-3.3, 8.3) | 1.6 (-1.6, 4.9) | 10.9* (6.0, 16.1) | 7.4* (0.7, 14.6) | 9.7* (4.3, 15.4) | 6.4* (4.9, 7.9) | 5.7* (4.0, 7.4) | 6.1*  (4.8, 7.5) |

Abbreviations: AAPC, average annual percent change; CI, confidence interval; KPSC, Kaiser Permanente Southern California

*denotes p<0.05.

Supplemental Table 2. Age-adjusted mortality rates (per 100,000 person-years) with heart failure as the underlying cause of death by sex among adults ≥65 years of age in Kaiser Permanente Southern California, California, and the United States, 2001-2017

| Year | KPSC | | | California | | | United States | | |
| --- | --- | --- | --- | --- | --- | --- | --- | --- | --- |
|  | Male | Female | Overall | Male | Female | Overall | Male | Female | Overall |
| 2001 | 91.8 | 44.2 | 64.0 | 96.9 | 83.9 | 89.0 | 157.1 | 145.2 | 150.6 |
| 2002 | 121.9 | 71.6 | 92.1 | 96.6 | 86.6 | 90.6 | 155.1 | 142.1 | 147.7 |
| 2003 | 119.0 | 88.5 | 100.5 | 110.3 | 94.3 | 100.7 | 156.3 | 141.1 | 147.5 |
| 2004 | 94.5 | 84.8 | 88.8 | 104.6 | 93.7 | 98.0 | 152.4 | 138.8 | 144.6 |
| 2005 | 112.0 | 95.9 | 102.7 | 107.7 | 99.9 | 103.4 | 152.7 | 140.5 | 145.8 |
| 2006 | 109.8 | 100.9 | 104.5 | 111.9 | 106.7 | 109.3 | 152.8 | 139.4 | 145.2 |
| 2007 | 99.7 | 89.3 | 94.4 | 101.6 | 89.6 | 94.5 | 142.0 | 126.3 | 132.9 |
| 2008 | 97.2 | 93.9 | 96.0 | 104.6 | 93.0 | 97.9 | 137.8 | 124.3 | 130.2 |
| 2009 | 128.0 | 91.6 | 106.7 | 107.4 | 89.5 | 96.8 | 137.3 | 118.5 | 126.3 |
| 2010 | 111.7 | 75.4 | 90.1 | 105.3 | 90.2 | 96.3 | 139.6 | 118.5 | 127.2 |
| 2011 | 125.2 | 98.5 | 108.8 | 112.4 | 95.1 | 102.1 | 136.1 | 116.4 | 124.5 |
| 2012 | 116.2 | 78.2 | 93.5 | 110.9 | 86.1 | 96.2 | 138.9 | 115.9 | 125.4 |
| 2013 | 106.9 | 85.7 | 94.6 | 109.7 | 90.5 | 98.1 | 146.5 | 121.4 | 131.7 |
| 2014 | 100.7 | 67.3 | 81.0 | 108.5 | 91.8 | 99.0 | 150.9 | 124.3 | 135.4 |
| 2015 | 113.7 | 93.7 | 102.1 | 123.1 | 99.5 | 109.4 | 162.5 | 133.2 | 145.4 |
| 2016 | 138.7 | 94.2 | 112.6 | 132.7 | 103.8 | 116.0 | 166.3 | 134.6 | 147.9 |
| 2017 | 134.0 | 107.4 | 119.4 | 135.8 | 108.1 | 119.8 | 167.2 | 135.0 | 148.4 |
| AAPC (95% CI) | | | | | | | | | |
| Overall | 1.3* (0.1, 2.5) | 5.3 (-0.3, 11.3) | 1.3* (0.0, 2.5) | 2.0*  (1.1, 2.9) | 1.6  (-0.1, 3.3) | 1.5* (0.2, 2.8) | 0.4  (-0.1, 0.9) | -0.3 (-1.1, 0.5) | -0.1  (-0.6, 0.4) |
| 2001-2011 | 1.3* (0.1, 2.5) | 6.1 (-2.2, 15.1) | 1.3* (0.0, 2.5) | 0.7*  (0.1, 1.4) | 0.6  (-1.6, 2.9) | 0.3 (-0.5, 1.1) | -1.6*  (-2.2, -1.1) | -2.3* (-3.4, -1.2) | -2.1*  (-2.7, -1.5) |
| 2011-2017 | 1.3* (0.1, 2.5) | 4.1 (-1.0, 9.3) | 1.3* (0.0, 2.5) | 4.1*  (1.9, 6.3) | 3.3*  (1.0, 5.6) | 3.5* (0.5, 6.6) | 3.9*  (2.8, 5.1) | 3.2* (2.1, 4.4) | 3.4*  (2.1, 4.6) |

Abbreviations: AAPC, average annual percent change; CI, confidence interval; KPSC, Kaiser Permanente Southern California

*denotes p<0.05.

Supplemental Table 3. Age-adjusted mortality rates (per 100,000 person-years) with heart failure as the underlying cause of death by sex and race/ethnicity among ≥ 45 years of age in Kaiser Permanente Southern California members, California, and the United States, 2001-2017

|  |  | **White** | | | **Hispanic** | | | **Black** | | | **Asian/Pacific Islander** | | |
| --- | --- | --- | --- | --- | --- | --- | --- | --- | --- | --- | --- | --- | --- |
| KPSC | Year | Male | Female | Overall | Male | Female | Overall | Male | Female | Overall | Male | Female | Overall |
|  | 2001 | 35.8 | 17.7 | 25.4 | 25.0 | 15.5 | 19.5 | 30.8 | 7.2 | 16.5 | 43.0 | 2.9 | 21.3 |
|  | 2002 | 43.2 | 28.2 | 34.3 | 49.2 | 31.8 | 39.3 | 90.4 | 19.7 | 46.7 | 14.2 | 24.1 | 19.6 |
|  | 2003 | 45.3 | 30.6 | 36.7 | 63.2 | 45.4 | 51.1 | 44.0 | 26.2 | 33.6 | 17.8 | 31.8 | 25.3 |
|  | 2004 | 40.8 | 33.4 | 36.5 | 21.7 | 19.2 | 20.7 | 26.7 | 37.0 | 33.0 | 9.8 | 22.3 | 16.6 |
|  | 2005 | 47.2 | 38.0 | 42.0 | 21.0 | 32.1 | 27.6 | 44.4 | 40.6 | 42.6 | 32.7 | 0.0 | 14.4 |
|  | 2006 | 46.7 | 40.4 | 42.8 | 26.6 | 32.7 | 31.1 | 40.0 | 48.2 | 46.0 | 16.6 | 19.5 | 18.1 |
|  | 2007 | 42.5 | 36.0 | 39.1 | 20.2 | 20.2 | 20.7 | 46.4 | 33.1 | 38.7 | 13.9 | 16.8 | 15.4 |
|  | 2008 | 38.0 | 39.3 | 39.1 | 32.9 | 34.4 | 34.3 | 62.1 | 27.5 | 40.2 | 6.0 | 8.7 | 7.5 |
|  | 2009 | 52.9 | 36.7 | 43.3 | 36.4 | 34.5 | 35.9 | 36.9 | 21.6 | 27.7 | 27.6 | 21.4 | 24.3 |
|  | 2010 | 45.7 | 30.4 | 36.4 | 31.7 | 19.5 | 24.6 | 47.6 | 32.6 | 38.9 | 17.4 | 22.4 | 20.9 |
|  | 2011 | 48.8 | 39.7 | 43.2 | 44.5 | 31.4 | 37.0 | 53.3 | 43.5 | 47.3 | 39.7 | 15.4 | 25.3 |
|  | 2012 | 46.4 | 32.5 | 38.2 | 45.5 | 21.4 | 31.3 | 38.6 | 27.4 | 31.7 | 21.9 | 13.7 | 17.1 |
|  | 2013 | 40.0 | 33.0 | 36.1 | 39.2 | 28.5 | 32.9 | 60.9 | 33.9 | 44.1 | 28.6 | 27.2 | 27.9 |
|  | 2014 | 39.8 | 23.8 | 30.6 | 33.6 | 21.9 | 26.7 | 45.6 | 35.3 | 38.6 | 24.4 | 20.9 | 22.5 |
|  | 2015 | 50.9 | 36.5 | 42.9 | 26.9 | 24.8 | 25.5 | 64.7 | 48.9 | 54.0 | 10.5 | 24.2 | 18.3 |
|  | 2016 | 57.4 | 41.1 | 47.9 | 36.4 | 25.4 | 30.1 | 78.4 | 39.4 | 53.1 | 33.2 | 23.4 | 27.5 |
|  | 2017 | 57.3 | 47.1 | 52.0 | 39.2 | 25.4 | 31.4 | 77.5 | 38.3 | 53.2 | 18.0 | 29.5 | 24.8 |
|  | | | | | | | | | | | | | |
| California | Year | Male | Female | Overall | Male | Female | Overall | Male | Female | Overall | Male | Female | Overall |
|  | 2001 | 40.4 | 34.4 | 36.8 | 24.8 | 24.8 | 25.0 | 47.3 | 32.3 | 37.8 | 20.3 | 15.0 | 17.2 |
|  | 2002 | 40.0 | 35.7 | 37.5 | 26.9 | 24.9 | 26.0 | 50.7 | 34.3 | 41.6 | 19.8 | 15.7 | 17.3 |
|  | 2003 | 45.5 | 38.7 | 41.6 | 37.3 | 27.9 | 31.6 | 49.9 | 37.6 | 41.7 | 22.5 | 18.1 | 19.8 |
|  | 2004 | 44.9 | 38.7 | 41.2 | 29.0 | 27.6 | 28.4 | 44.8 | 39.7 | 42.2 | 16.2 | 16.8 | 16.6 |
|  | 2005 | 46.2 | 41.1 | 43.3 | 31.7 | 31.4 | 31.8 | 47.5 | 43.2 | 45.3 | 15.0 | 15.8 | 15.5 |
|  | 2006 | 48.6 | 44.7 | 46.6 | 34.1 | 33.7 | 34.3 | 54.0 | 47.8 | 50.1 | 18.1 | 18.3 | 18.2 |
|  | 2007 | 44.1 | 38.4 | 40.8 | 29.3 | 25.7 | 27.1 | 50.4 | 38.6 | 43.3 | 14.6 | 12.8 | 13.5 |
|  | 2008 | 45.2 | 38.5 | 41.4 | 31.0 | 28.2 | 29.3 | 52.2 | 42.7 | 46.8 | 16.8 | 17.2 | 17.0 |
|  | 2009 | 46.0 | 37.5 | 41.1 | 35.0 | 27.9 | 30.8 | 45.7 | 38.9 | 42.4 | 17.9 | 14.3 | 15.7 |
|  | 2010 | 45.5 | 37.5 | 40.9 | 32.7 | 27.8 | 29.8 | 42.5 | 40.2 | 41.3 | 17.9 | 17.5 | 17.8 |
|  | 2011 | 47.6 | 39.9 | 43.0 | 39.1 | 31.6 | 34.8 | 47.5 | 43.5 | 45.8 | 19.4 | 15.7 | 17.3 |
|  | 2012 | 48.4 | 36.0 | 41.2 | 34.4 | 27.3 | 30.3 | 51.4 | 43.3 | 46.8 | 19.0 | 15.7 | 17.1 |
|  | 2013 | 48.3 | 37.7 | 42.1 | 32.2 | 31.6 | 31.9 | 56.8 | 42.6 | 48.5 | 21.1 | 16.1 | 18.2 |
|  | 2014 | 48.5 | 39.9 | 43.7 | 34.2 | 28.7 | 31.2 | 57.9 | 48.1 | 52.1 | 18.8 | 15.5 | 17.0 |
|  | 2015 | 56.3 | 44.3 | 49.6 | 37.8 | 31.8 | 34.5 | 61.4 | 45.3 | 52.3 | 22.7 | 15.9 | 18.6 |
|  | 2016 | 59.8 | 44.1 | 51.0 | 44.3 | 35.1 | 39.1 | 64.4 | 53.4 | 58.1 | 23.3 | 20.5 | 21.8 |
|  | 2017 | 60.4 | 46.9 | 52.8 | 46.6 | 34.1 | 39.5 | 70.2 | 53.0 | 60.5 | 25.7 | 20.8 | 23.0 |
|  | | | | | | | | | | | | | |
| United States | Year | Male | Female | Overall | Male | Female | Overall | Male | Female | Overall | Male | Female | Overall |
|  | 2001 | 62.6 | 56.6 | 59.4 | 31.2 | 30.0 | 30.7 | 70.3 | 59.7 | 63.8 | 20.1 | 18.0 | 18.9 |
|  | 2002 | 61.5 | 55.2 | 58.0 | 32.5 | 29.5 | 30.8 | 68.7 | 59.8 | 63.7 | 22.6 | 18.7 | 20.2 |
|  | 2003 | 61.9 | 54.8 | 57.8 | 35.8 | 30.5 | 32.9 | 69.8 | 60.2 | 64.1 | 21.9 | 19.3 | 20.4 |
|  | 2004 | 60.4 | 54.4 | 57.0 | 31.8 | 28.4 | 29.9 | 70.1 | 57.2 | 62.2 | 20.8 | 17.8 | 19.1 |
|  | 2005 | 60.6 | 54.8 | 57.3 | 34.4 | 30.5 | 32.2 | 68.5 | 60.8 | 64.3 | 20.6 | 18.4 | 19.3 |
|  | 2006 | 61.1 | 54.7 | 57.6 | 34.5 | 30.0 | 32.0 | 69.2 | 57.3 | 62.1 | 20.5 | 19.3 | 19.9 |
|  | 2007 | 56.6 | 49.4 | 52.5 | 34.1 | 28.8 | 31.0 | 66.9 | 54.9 | 59.8 | 17.5 | 15.8 | 16.5 |
|  | 2008 | 55.2 | 48.6 | 51.6 | 31.7 | 29.3 | 30.4 | 65.7 | 51.8 | 57.4 | 18.3 | 18.2 | 18.3 |
|  | 2009 | 55.3 | 46.7 | 50.3 | 31.6 | 26.9 | 29.0 | 61.2 | 48.9 | 54.0 | 18.9 | 18.1 | 18.4 |
|  | 2010 | 56.1 | 46.7 | 50.7 | 32.2 | 25.6 | 28.3 | 63.8 | 49.6 | 55.4 | 20.1 | 18.4 | 19.2 |
|  | 2011 | 54.9 | 46.1 | 49.8 | 31.7 | 26.2 | 28.6 | 59.5 | 48.8 | 53.3 | 20.9 | 16.5 | 18.2 |
|  | 2012 | 56.0 | 45.9 | 50.2 | 33.3 | 26.1 | 29.2 | 62.6 | 48.7 | 54.5 | 21.4 | 18.0 | 19.4 |
|  | 2013 | 59.8 | 48.3 | 53.1 | 31.5 | 27.0 | 28.9 | 67.0 | 52.4 | 58.4 | 23.0 | 18.4 | 20.4 |
|  | 2014 | 61.9 | 49.9 | 55.0 | 32.0 | 26.4 | 28.9 | 69.8 | 53.7 | 60.2 | 21.9 | 19.3 | 20.5 |
|  | 2015 | 66.6 | 53.5 | 59.1 | 36.3 | 29.2 | 32.2 | 76.5 | 57.1 | 65.0 | 24.1 | 18.5 | 20.8 |
|  | 2016 | 68.5 | 54.0 | 60.3 | 38.6 | 30.6 | 34.1 | 77.0 | 61.1 | 67.6 | 24.3 | 21.2 | 22.5 |
|  | 2017 | 69.1 | 54.4 | 60.7 | 38.2 | 29.5 | 33.3 | 79.9 | 60.4 | 68.3 | 24.8 | 20.7 | 22.6 |

Abbreviations: KPSC, Kaiser Permanente Southern California

Supplemental Table 4. Age-adjusted mortality rates (per 100,000 person-years) with heart failure as the underlying cause of death among adults 45-64 years of age by sex and race/ethnicity among Kaiser Permanente Southern California members, California, and the United States, 2001-2017

|  |  | **White** | | | **Hispanic** | | | **Black** | | | **Asian/Pacific Islander** | | |
| --- | --- | --- | --- | --- | --- | --- | --- | --- | --- | --- | --- | --- | --- |
| KPSC | Year | Male | Female | Overall | Male | Female | Overall | Male | Female | Overall | Male | Female | Overall |
|  | 2001 | 0.6 | 0.0 | 0.3 | 1.5 | 0.0 | 0.7 | 8.9 | 4.4 | 6.3 | 0.0 | 0.0 | 0.0 |
|  | 2002 | 2.3 | 1.3 | 1.8 | 1.4 | 0.0 | 0.7 | 16.5 | 8.4 | 11.9 | 0.0 | 0.0 | 0.0 |
|  | 2003 | 1.6 | 0.5 | 1.0 | 3.8 | 0.0 | 1.8 | 8.2 | 0.0 | 3.5 | 0.0 | 0.0 | 0.0 |
|  | 2004 | 3.7 | 0.5 | 2.0 | 1.2 | 1.1 | 1.1 | 2.7 | 8.1 | 5.8 | 0.0 | 0.0 | 0.0 |
|  | 2005 | 2.2 | 0.9 | 1.5 | 2.2 | 0.0 | 1.1 | 0.0 | 5.7 | 3.3 | 5.3 | 0.0 | 2.3 |
|  | 2006 | 2.4 | 3.7 | 3.1 | 1.9 | 0.0 | 0.9 | 13.5 | 4.0 | 8.0 | 0.0 | 0.0 | 0.0 |
|  | 2007 | 3.2 | 0.4 | 1.8 | 0.0 | 0.0 | 0.0 | 7.4 | 0.0 | 3.1 | 0.0 | 0.0 | 0.0 |
|  | 2008 | 1.6 | 1.2 | 1.4 | 4.2 | 1.5 | 2.8 | 10.4 | 5.6 | 7.6 | 0.0 | 3.9 | 2.2 |
|  | 2009 | 1.1 | 1.0 | 1.1 | 3.2 | 0.0 | 1.5 | 5.1 | 0.0 | 2.2 | 0.0 | 0.0 | 0.0 |
|  | 2010 | 1.1 | 1.4 | 1.2 | 0.0 | 2.1 | 1.1 | 15.1 | 0.0 | 6.4 | 1.9 | 1.5 | 1.7 |
|  | 2011 | 3.4 | 1.1 | 2.2 | 1.4 | 0.6 | 1.0 | 9.2 | 5.2 | 6.9 | 1.8 | 0.0 | 0.8 |
|  | 2012 | 2.0 | 0.4 | 1.1 | 2.1 | 1.2 | 1.6 | 4.2 | 2.1 | 3.0 | 1.8 | 0.0 | 0.8 |
|  | 2013 | 1.8 | 0.7 | 1.3 | 1.9 | 2.4 | 2.2 | 14.4 | 4.6 | 8.8 | 5.2 | 2.7 | 3.8 |
|  | 2014 | 0.8 | 0.0 | 0.4 | 2.4 | 1.2 | 1.8 | 0.0 | 2.1 | 1.2 | 5.0 | 0.0 | 2.2 |
|  | 2015 | 2.9 | 0.3 | 1.6 | 2.8 | 2.0 | 2.4 | 1.9 | 4.9 | 3.6 | 1.6 | 3.0 | 2.4 |
|  | 2016 | 1.7 | 2.4 | 2.0 | 2.0 | 1.9 | 2.0 | 16.6 | 8.3 | 11.8 | 1.5 | 1.2 | 1.3 |
|  | 2017 | 4.1 | 1.0 | 2.5 | 1.4 | 0.9 | 1.2 | 12.9 | 1.4 | 6.3 | 0.0 | 1.1 | 0.6 |
|  | | | | | | | | | | | | | |
| California | Year | Male | Female | Overall | Male | Female | Overall | Male | Female | Overall | Male | Female | Overall |
|  | 2001 | 3.2 | 2.2 | 2.7 | NA | NA | 1.9 | NA | NA | 6.6 | NA | NA | NA |
|  | 2002 | 3.4 | 2.1 | 2.8 | NA | NA | 2.2 | 11.0 | NA | 8.2 | NA | NA | NA |
|  | 2003 | 4.0 | 2.2 | 3.1 | 3.7 | NA | 2.8 | 9.1 | NA | 7.8 | NA | NA | NA |
|  | 2004 | 3.6 | 2.2 | 2.9 | 2.8 | 2.4 | 2.6 | 12.5 | NA | 9.4 | NA | NA | NA |
|  | 2005 | 3.5 | 1.9 | 2.7 | NA | NA | 2.0 | 10.5 | NA | 8.2 | NA | NA | NA |
|  | 2006 | 5.3 | 3.1 | 4.2 | 2.6 | 2.5 | 2.5 | 14.9 | 8.8 | 11.7 | NA | NA | NA |
|  | 2007 | 3.5 | 2.2 | 2.8 | 2.9 | NA | 2.3 | 10.1 | NA | 8.1 | NA | NA | NA |
|  | 2008 | 3.5 | 2.3 | 2.9 | 3.7 | 1.8 | 2.7 | 11.5 | NA | 8.3 | NA | NA | NA |
|  | 2009 | 3.3 | 1.9 | 2.6 | 3.8 | NA | 2.6 | 12.1 | 7.4 | 9.7 | NA | NA | NA |
|  | 2010 | 3.0 | 1.6 | 2.3 | 2.1 | 1.9 | 2.0 | 12.1 | 7.4 | 9.7 | NA | NA | 1.7 |
|  | 2011 | 2.9 | 2.0 | 2.4 | 3.8 | 2.2 | 3.0 | 12.7 | NA | 8.6 | NA | NA | NA |
|  | 2012 | 3.6 | 1.7 | 2.6 | 3.0 | 1.7 | 2.3 | 12.9 | 7.9 | 10.4 | NA | NA | 1.6 |
|  | 2013 | 4.4 | 2.3 | 3.4 | 2.9 | 2.1 | 2.5 | 13.4 | 6.1 | 9.6 | 3.2 | NA | 1.8 |
|  | 2014 | 4.9 | 2.8 | 3.9 | 4.6 | 2.5 | 3.5 | 11.2 | 9.8 | 10.5 | NA | NA | 1.8 |
|  | 2015 | 6.3 | 3.1 | 4.7 | 5.0 | 1.9 | 3.5 | 14.9 | 10.4 | 12.6 | 3.0 | NA | 1.9 |
|  | 2016 | 6.4 | 3.1 | 4.8 | 4.7 | 2.0 | 3.3 | 17.5 | 12.6 | 15.0 | 2.8 | NA | 1.8 |
|  | 2017 | 6.2 | 2.2 | 4.2 | 5.5 | 2.9 | 4.2 | 20.8 | 10.7 | 15.6 | 3.8 | NA | 2.4 |
|  | | | | | | | | | | | | | |
| United States | Year | Male | Female | Overall | Male | Female | Overall | Male | Female | Overall | Male | Female | Overall |
|  | 2001 | 5.8 | 3.6 | 4.7 | 3.5 | 2.5 | 3.0 | 13.5 | 10.5 | 11.8 | NA | NA | 1.2 |
|  | 2002 | 5.4 | 3.5 | 4.4 | 3.0 | 2.2 | 2.5 | 14.5 | 9.6 | 11.8 | NA | NA | 1.3 |
|  | 2003 | 5.3 | 3.4 | 4.3 | 3.9 | 2.2 | 3.0 | 15.4 | 9.9 | 12.4 | NA | NA | 0.9 |
|  | 2004 | 5.0 | 3.4 | 4.2 | 3.4 | 1.9 | 2.6 | 15.4 | 9.6 | 12.3 | 2.0 | NA | 1.4 |
|  | 2005 | 5.2 | 3.4 | 4.3 | 2.5 | 2.4 | 2.4 | 15.4 | 9.9 | 12.4 | 1.7 | NA | 1.3 |
|  | 2006 | 5.7 | 3.4 | 4.5 | 3.2 | 2.1 | 2.6 | 15.8 | 10.1 | 12.8 | 2.0 | NA | 1.4 |
|  | 2007 | 5.1 | 3.2 | 4.1 | 3.6 | 2.0 | 2.8 | 15.7 | 9.3 | 12.2 | NA | NA | 0.8 |
|  | 2008 | 5.3 | 3.0 | 4.1 | 3.8 | 2.2 | 3.0 | 15.3 | 9.0 | 11.9 | 1.5 | 1.3 | 1.4 |
|  | 2009 | 5.4 | 3.0 | 4.1 | 4.1 | 2.0 | 3.0 | 14.9 | 9.2 | 11.8 | 1.9 | 1.1 | 1.4 |
|  | 2010 | 5.2 | 3.0 | 4.1 | 2.8 | 2.0 | 2.4 | 14.6 | 8.3 | 11.2 | 1.8 | 1.2 | 1.5 |
|  | 2011 | 5.0 | 3.1 | 4.0 | 3.4 | 2.1 | 2.7 | 13.9 | 8.2 | 10.8 | 1.5 | NA | 1.2 |
|  | 2012 | 5.1 | 3.0 | 4.0 | 3.5 | 1.9 | 2.7 | 15.7 | 8.6 | 11.9 | 1.5 | 1.4 | 1.4 |
|  | 2013 | 5.9 | 3.3 | 4.5 | 3.6 | 2.1 | 2.8 | 17.0 | 9.8 | 13.1 | 2.9 | 1.0 | 1.9 |
|  | 2014 | 6.2 | 3.5 | 4.9 | 3.9 | 2.5 | 3.2 | 16.9 | 11.0 | 13.7 | 2.5 | 1.2 | 1.8 |
|  | 2015 | 6.7 | 3.8 | 5.2 | 4.6 | 1.9 | 3.2 | 19.6 | 10.6 | 14.7 | 2.6 | 1.1 | 1.8 |
|  | 2016 | 7.0 | 4.1 | 5.5 | 4.8 | 2.4 | 3.5 | 20.5 | 12.0 | 15.9 | 2.6 | 0.9 | 1.7 |
|  | 2017 | 7.3 | 3.8 | 5.5 | 5.1 | 2.5 | 3.8 | 21.0 | 12.7 | 16.5 | 2.9 | 1.0 | 1.9 |

Abbreviations: KPSC, Kaiser Permanente Southern California

Supplemental Table 5. Age-adjusted mortality rates (per 100,000 person-years) with heart failure as the underlying cause of death among adults ≥ 65 years of age by sex and race/ethnicity among Kaiser Permanente Southern California members, California, and the United States, 2001-2017

|  |  | **White** | | | **Hispanic** | | | **Black** | | | **Asian/Pacific Islander** | | |
| --- | --- | --- | --- | --- | --- | --- | --- | --- | --- | --- | --- | --- | --- |
| KPSC | Year | Male | Female | Overall | Male | Female | Overall | Male | Female | Overall | Male | Female | Overall |
|  | 2001 | 97.8 | 48.9 | 69.5 | 66.3 | 42.6 | 52.5 | 69.4 | 12.1 | 34.4 | 118.4 | 8.0 | 58.8 |
|  | 2002 | 115.2 | 75.4 | 91.5 | 133.3 | 87.6 | 107.1 | 220.3 | 39.6 | 107.8 | 39.2 | 66.5 | 54.0 |
|  | 2003 | 122.1 | 83.6 | 99.5 | 167.6 | 125.1 | 137.6 | 107.1 | 72.2 | 86.5 | 49.0 | 87.7 | 69.7 |
|  | 2004 | 106.1 | 91.2 | 97.0 | 57.7 | 51.1 | 55.0 | 69.0 | 87.9 | 80.9 | 27.0 | 61.4 | 45.9 |
|  | 2005 | 126.2 | 103.2 | 113.0 | 54.0 | 88.6 | 74.3 | 122.5 | 102.0 | 111.6 | 80.7 | 0.0 | 35.5 |
|  | 2006 | 124.6 | 104.9 | 112.5 | 70.0 | 90.2 | 84.1 | 86.7 | 125.9 | 112.8 | 45.7 | 53.8 | 49.8 |
|  | 2007 | 111.7 | 98.6 | 104.8 | 55.6 | 55.7 | 57.1 | 114.8 | 91.3 | 101.4 | 38.4 | 46.4 | 42.4 |
|  | 2008 | 102.0 | 106.0 | 105.2 | 83.3 | 92.3 | 89.6 | 153.0 | 66.2 | 97.3 | 16.6 | 17.1 | 16.7 |
|  | 2009 | 143.8 | 99.3 | 117.5 | 94.7 | 95.2 | 96.3 | 92.8 | 59.5 | 72.5 | 76.1 | 59.0 | 66.9 |
|  | 2010 | 124.1 | 81.4 | 98.2 | 87.5 | 50.1 | 66.0 | 104.8 | 89.8 | 95.9 | 44.6 | 59.1 | 54.7 |
|  | 2011 | 128.4 | 107.5 | 115.2 | 120.3 | 85.5 | 100.1 | 130.8 | 110.9 | 118.3 | 106.2 | 42.4 | 68.3 |
|  | 2012 | 124.4 | 89.1 | 103.5 | 121.9 | 57.0 | 83.4 | 99.1 | 71.8 | 82.3 | 57.2 | 37.8 | 45.7 |
|  | 2013 | 107.1 | 89.8 | 97.2 | 104.6 | 74.3 | 86.9 | 142.5 | 85.4 | 106.2 | 69.6 | 70.3 | 70.3 |
|  | 2014 | 108.3 | 65.7 | 83.8 | 88.3 | 58.2 | 70.4 | 125.7 | 93.6 | 104.4 | 58.5 | 57.6 | 58.1 |
|  | 2015 | 135.2 | 100.0 | 115.4 | 69.3 | 64.9 | 66.2 | 174.9 | 126.2 | 142.6 | 26.2 | 61.5 | 46.3 |
|  | 2016 | 155.2 | 109.0 | 128.4 | 96.7 | 66.6 | 79.7 | 186.9 | 94.0 | 125.6 | 88.9 | 62.4 | 73.4 |
|  | 2017 | 150.9 | 128.0 | 138.8 | 105.6 | 68.3 | 84.6 | 191.1 | 103.3 | 135.7 | 49.7 | 79.3 | 67.4 |
|  | | | | | | | | | | | | | |
| California | Year | Male | Female | Overall | Male | Female | Overall | Male | Female | Overall | Male | Female | Overall |
|  | 2001 | 105.7 | 91.0 | 96.7 | 64.6 | 65.5 | 65.4 | 115.7 | 80.3 | 92.8 | 53.8 | 39.9 | 45.5 |
|  | 2002 | 104.2 | 94.6 | 98.4 | 70.0 | 65.2 | 67.8 | 120.4 | 84.6 | 100.4 | 51.4 | 41.3 | 45.2 |
|  | 2003 | 118.5 | 102.7 | 109.2 | 96.4 | 73.4 | 82.3 | 121.6 | 92.1 | 101.3 | 60.1 | 47.6 | 52.6 |
|  | 2004 | 117.4 | 102.9 | 108.5 | 75.0 | 72.0 | 73.7 | 101.6 | 97.5 | 99.9 | 42.9 | 43.9 | 43.6 |
|  | 2005 | 121.3 | 109.8 | 114.8 | 83.7 | 83.2 | 84.3 | 112.5 | 108.2 | 110.4 | 38.4 | 42.5 | 40.8 |
|  | 2006 | 124.8 | 117.8 | 121.1 | 89.4 | 88.7 | 90.2 | 122.8 | 116.5 | 117.5 | 47.7 | 48.7 | 48.3 |
|  | 2007 | 115.4 | 102.0 | 107.6 | 75.7 | 67.6 | 70.7 | 121.1 | 95.7 | 105.3 | 38.5 | 34.6 | 36.2 |
|  | 2008 | 118.4 | 102.0 | 109.1 | 78.8 | 74.6 | 76.1 | 123.6 | 108.4 | 114.4 | 44.9 | 45.2 | 45.1 |
|  | 2009 | 120.8 | 100.1 | 108.6 | 89.9 | 74.4 | 80.5 | 104.7 | 94.2 | 99.9 | 46.8 | 38.5 | 41.8 |
|  | 2010 | 120.0 | 100.7 | 108.6 | 86.5 | 73.1 | 78.6 | 95.8 | 97.9 | 96.7 | 45.8 | 45.8 | 46.1 |
|  | 2011 | 126.2 | 106.5 | 114.4 | 101.1 | 83.2 | 90.6 | 108.7 | 111.7 | 111.2 | 50.6 | 42.4 | 45.7 |
|  | 2012 | 127.1 | 96.4 | 108.9 | 89.7 | 72.3 | 79.4 | 119.0 | 105.5 | 110.8 | 49.7 | 40.6 | 44.3 |
|  | 2013 | 125.4 | 99.9 | 110.1 | 83.7 | 83.5 | 83.5 | 133.1 | 106.6 | 116.8 | 52.4 | 43.1 | 47.1 |
|  | 2014 | 125.1 | 105.1 | 113.7 | 86.2 | 74.8 | 79.9 | 140.0 | 115.5 | 125.3 | 47.6 | 40.6 | 43.9 |
|  | 2015 | 144.2 | 116.8 | 128.4 | 95.4 | 84.2 | 89.0 | 143.0 | 106.6 | 122.0 | 57.4 | 41.9 | 48.0 |
|  | 2016 | 153.8 | 116.1 | 132.1 | 113.9 | 93.3 | 101.9 | 146.8 | 125.3 | 133.8 | 59.2 | 54.9 | 57.0 |
|  | 2017 | 155.7 | 125.4 | 138.2 | 118.8 | 89.0 | 101.6 | 157.0 | 127.4 | 139.2 | 64.2 | 55.1 | 59.2 |
|  | | | | | | | | | | | | | |
| United States | Year | Male | Female | Overall | Male | Female | Overall | Male | Female | Overall | Male | Female | Overall |
|  | 2001 | 162.4 | 149.7 | 155.5 | 80.1 | 78.2 | 79.3 | 170.1 | 146.1 | 155.2 | 53.0 | 48.0 | 50.1 |
|  | 2002 | 160.1 | 146.2 | 152.2 | 84.4 | 77.3 | 80.5 | 163.9 | 148.1 | 154.8 | 60.2 | 49.3 | 53.4 |
|  | 2003 | 161.4 | 145.2 | 152.0 | 92.1 | 80.3 | 85.3 | 165.4 | 148.6 | 154.9 | 58.6 | 51.7 | 54.6 |
|  | 2004 | 157.8 | 144.0 | 149.9 | 81.7 | 74.8 | 77.9 | 166.4 | 140.8 | 149.9 | 53.8 | 47.5 | 50.1 |
|  | 2005 | 158.1 | 145.1 | 150.5 | 90.4 | 80.0 | 84.5 | 161.8 | 150.3 | 155.7 | 53.8 | 48.9 | 51.0 |
|  | 2006 | 158.5 | 144.9 | 150.8 | 89.3 | 79.1 | 83.7 | 162.9 | 140.2 | 148.7 | 53.1 | 51.6 | 52.3 |
|  | 2007 | 147.0 | 130.6 | 137.6 | 87.6 | 76.0 | 80.6 | 156.9 | 135.0 | 143.4 | 46.4 | 42.4 | 44.1 |
|  | 2008 | 142.7 | 128.8 | 135.0 | 80.8 | 76.8 | 78.6 | 154.4 | 127.0 | 137.5 | 47.7 | 47.8 | 47.8 |
|  | 2009 | 143.0 | 123.4 | 131.6 | 80.0 | 70.8 | 74.8 | 142.4 | 118.6 | 128.0 | 48.7 | 47.9 | 48.3 |
|  | 2010 | 145.5 | 123.5 | 132.6 | 84.0 | 67.1 | 73.9 | 150.1 | 122.1 | 133.0 | 52.2 | 48.6 | 50.3 |
|  | 2011 | 142.6 | 121.7 | 130.4 | 81.6 | 68.4 | 74.0 | 139.7 | 120.3 | 128.0 | 55.0 | 44.1 | 48.3 |
|  | 2012 | 145.4 | 121.4 | 131.4 | 85.6 | 68.7 | 75.8 | 145.1 | 119.1 | 129.4 | 56.4 | 47.1 | 50.9 |
|  | 2013 | 154.5 | 127.3 | 138.5 | 80.6 | 70.7 | 74.8 | 154.9 | 127.3 | 137.9 | 58.3 | 49.0 | 52.8 |
|  | 2014 | 159.6 | 131.3 | 143.2 | 81.5 | 68.5 | 74.0 | 162.8 | 128.7 | 141.8 | 56.0 | 51.1 | 53.4 |
|  | 2015 | 171.8 | 140.9 | 153.9 | 91.9 | 77.2 | 83.2 | 176.4 | 138.7 | 153.6 | 62.0 | 49.2 | 54.2 |
|  | 2016 | 176.5 | 141.7 | 156.5 | 98.0 | 80.2 | 87.7 | 176.4 | 147.4 | 158.6 | 62.2 | 56.8 | 59.0 |
|  | 2017 | 177.6 | 143.3 | 157.7 | 96.2 | 77.0 | 85.1 | 183.6 | 144.0 | 159.1 | 63.4 | 55.2 | 58.9 |

Abbreviations: KPSC, Kaiser Permanente Southern California
